# Sustained expression of MCP-1 by low wall shear stress loading concomitant with turbulent flow on endothelial cells of intracranial aneurysm

**DOI:** 10.1186/s40478-016-0318-3

**Published:** 2016-05-09

**Authors:** Tomohiro Aoki, Kimiko Yamamoto, Miyuki Fukuda, Yuji Shimogonya, Shunichi Fukuda, Shuh Narumiya

**Affiliations:** Center for Innovation in Immunoregulation Technology and Therapeutics, Kyoto University Graduate School of Medicine, Konoe-cho Yoshida, Sakyo-ku, Kyoto, Kyoto 606-8501 Japan; Core Research for Evolutional Science and Technology, Medical Innovation Center, Kyoto University Graduate School of Medicine, 53 Kawahara-cho Shogoin, Sakyo-ku, Kyoto City, Kyoto 606-8507 Japan; System Physiology, Department of Biomedical Engineering, Graduate School of Medicine, The University of Tokyo, 7-3-1 Hongo, Bunkyo-ku, Tokyo, 113-0033 Japan; Department of Neurosurgery, Kyoto University Graduate School of Medicine, 54 Kawahara-cho Shogoin, Sakyo-ku, Kyoto City, Kyoto 606-8507 Japan; Frontier Research Institute for Interdisciplinary Sciences, Tohoku University, 6-3 Aramaki aza Aoba, Aoba-ku, Sendai City, Miyagi 980-8578 Japan; Department of Neurosurgery, National Hospital Organization Kyoto Medical Center, 1-1 Mukaihata-cho Fukakusa, Fushimi-ku, Kyoto City, Kyoto 612-8555 Japan

## Abstract

**Introduction:**

Enlargement of a pre-existing intracranial aneurysm is a well-established risk factor of rupture. Excessive low wall shear stress concomitant with turbulent flow in the dome of an aneurysm may contribute to progression and rupture. However, how stress conditions regulate enlargement of a pre-existing aneurysm remains to be elucidated.

**Results:**

Wall shear stress was calculated with 3D-computational fluid dynamics simulation using three cases of unruptured intracranial aneurysm. The resulting value, 0.017 Pa at the dome, was much lower than that in the parent artery. We loaded wall shear stress corresponding to the value and also turbulent flow to the primary culture of endothelial cells. We then obtained gene expression profiles by RNA sequence analysis. RNA sequence analysis detected hundreds of differentially expressed genes among groups. Gene ontology and pathway analysis identified signaling related with cell division/proliferation as overrepresented in the low wall shear stress–loaded group, which was further augmented by the addition of turbulent flow. Moreover, expression of some chemoattractants for inflammatory cells, including MCP-1, was upregulated under low wall shear stress with concomitant turbulent flow. We further examined the temporal sequence of expressions of factors identified in an *in vitro* study using a rat model. No proliferative cells were detected, but MCP-1 expression was induced and sustained in the endothelial cell layer.

**Conclusions:**

Low wall shear stress concomitant with turbulent flow contributes to sustained expression of MCP-1 in endothelial cells and presumably plays a role in facilitating macrophage infiltration and exacerbating inflammation, which leads to enlargement or rupture.

## Introduction

As medical treatments have advanced, the outcomes for patients with various diseases have greatly improved. However, in cases of subarachnoid hemorrhage, this is not necessarily true. Subarachnoid hemorrhage often causes sudden death without a chance for therapeutic intervention, even in young patients, and its prognosis is still quite poor regardless of improvements in neurocritical care [1]. Intracranial aneurysm (IA), histologically characterized as a lesion with disrupted internal elastic lamina and excessive degeneration of media, is a major cause of subarachnoid hemorrhage [2, 3]. Considering the poor outcomes once after subarachnoid hemorrhage occurs [1] and the high incidence of IA in the general population [2], appropriate treatment to prevent rupture is crucial. However, there is no practicable medical treatment available for patients with IAs to prevent enlargement or rupture. The primary reason underlying this reality is that the detailed mechanisms regulating IA formation and progression remain to be elucidated.

Experimental studies using an animal model of IA have clarified the crucial role of persistent inflammation, presumably triggered by high wall shear stress (WSS) loaded on intracranial arterial walls at bifurcation sites and maintained/amplified by the formation of a positive feedback loop that includes the cyclooxygenase (COX)-2-prostaglandin (PG) E_2_-EP2-NF-κB cascade in endothelial cells (ECs) [4–6]. Computational fluid dynamic (CFD) analyses of human intracranial artery or IA lesions have supported this notion because high WSS can be detected at the prospective site of IA formation or the neck portion of IAs [7, 8]. Because the pharmacological inhibition of pro-inflammatory factors or genetic deletion of pro-inflammatory genes significantly suppresses not only the incidence but also the enlargement of IAs [4, 5, 9–11], long-lasting inflammation in intracranial arteries plays a role in both these steps. In terms of inflammation, therefore, the initiation and enlargement/progression of IAs share the same machinery. However, the hemodynamic status during these two steps is completely opposite. In the dome of growing IAs, the region of low WSS significantly overlaps with that of the enlarging portion [12, 13], suggesting the role of low WSS in the enlargement of IAs. Importantly, at the rupture point of IAs, the presence of low WSS and concomitant turbulent flow have also been demonstrated [14, 15], although there is a controversy regarding WSS status relating with the rupture of IAs [16]. However, the contribution of low WSS with or without concomitant turbulent flow loaded on the ECs of IA walls to enlargement and rupture remains to be elucidated. Considered with the fact that the target of treatment is of course pre-existing IAs and that a recent cohort study in Japan clearly demonstrated the positive correlation of the size of IAs with the annual risk of rupture [17], factors induced in ECs under low WSS condition may be good candidates for development of therapeutic drugs to prevent enlargement and presumably rupture of pre-existing IAs.

In the present study, we loaded WSS calculated from human cases by three-dimensional (3D) computational simulation on primary culture of ECs and examined the change in gene expression profiles and the temporal sequence of their expression in lesions.

## Materials and methods

### Data acquisition

Usage of 3D-computational tomography angiography (3D-CTA) data in the present study was approved by the review committee of the National Hospital Organization, Kyoto Medical Center in Kyoto, Japan.

Three patients with unruptured aneurysm were enrolled in the present study. In order to calculate WSS acting on the dome of human IAs and parent arteries, we employed three patient-specific, anatomically realistic arterial geometries of unruptured IAs. The geometries were segmented precisely from the volume data set of 3D-CTA using the Vascular Modeling Toolkit (VMTK) [18]. To apply CFD technique to these geometries, we generated high-quality computational meshes, including wall boundary-fitted layers, which were necessary to capture the steep velocity profiles near the wall boundary and to calculate WSS accurately, using the Mixed-Element Grid Generator in 3 Dimensions (MEGG3D) [19, 20]. A well-validated CFD solver, OpenFOAM, was used to perform blood flow analyses. In the CFD analyses, blood was treated as a Newtonian fluid and the wall was assumed to be rigid. We used patient-specific flow velocities measured with the ultrasound Doppler technique as the inlet boundary condition of the CFD analyses.

### Primary culture of endothelial cells from human carotid artery

The primary culture of ECs from human carotid artery was purchased from Cell Applications (San Diego, CA, USA). The characteristics of these cells were confirmed to be compatible with those of ECs in some methods, including immunohistochemistry [4].

### Loading shear stress

The primary culture of ECs, cultured on gelatin-coated glass slides, were loaded with a shear stress at 0.05 or 3.0 Pa according to the result from CFD analyses with a custom-made apparatus, as previously described [21]. After 24 h of shear stress loading, the cells were harvested. In addition, turbulent flow was loaded on ECs with a custom-made apparatus, as previously reported [22].

### RNA extraction and cap analysis of gene expression analysis

Using an RNeasy Plus Mini Kit (QIAGEN, Hilden, Germany), total RNA was prepared from ECs loaded on shear stress or kept in a static condition as a control; the quality was checked by an RNA analyzer. RNA sequence-based cap analysis of gene expression **(**CAGE) analysis, which was conducted by the Genome Network Analysis Support Facility (GeNAS) at RIKEN (http://www.osc.riken.jp/genas/), was employed to obtain the gene expression profile [23, 24]. Pathway and gene ontology (GO) analyses were performed with the Platform for Drug Discovery housed at the Data Analysis Center of the National Institute of Genetics (http://cell-innovation.nig.ac.jp/wiki2/tiki-index.php?page=5.+CAGE) using peak data obtained by the CAGE analysis.

### Quantitative real time PCR

Total RNA was prepared as described in the previous section and reverse-transcribed into cDNA using the High Capacity cDNA Reverse Transcription Kit (Life Technologies Corporation, Carlsbad, CA). Then, quantitative real time PCR (RT-PCR) was performed with the SYBR Premix Ex Taq II (Takara, Shiga, Japan) and Real Time System CFX96 (Bio-Rad Laboratories, Irvine, CA). β-actin was used as an internal control. For quantification, the second derivative maximum method was used for crossing point determination. Primer sets used are listed in Table 1.

**Table 1:** List of primer sets used in the present study

Gene name	Reference Sequence	forward primer (5′ → 3′)	reverse primer (5′ → 3′)
*ACTB*	NM_001101.3	CAT ACT CCT GCT TGC TGA TCC	GAT GCA GAA GGA GAT CAC TGC
*CCL2*	NM_002982.3	AGC TTC TTT GGG ACA CTT GC	ATA GCA GCC ACC TTC ATT CC
*CCNB*	NM_031966.3	GGA TCA GCT CCA TCT TCT GC	TTT GGT TGA TAC TGC CTC TCC
*CDCA7*	NM_031942.3	GAA ACT AAG CGG TTG GAG AGG	TTC TGG AGC ATC ACA GAA GG
*CDC20*	NM_001255.2	CAG AGC ACA CAT TCC AGA TGC	GTT CCT CTG CAG ACA TTC ACC
*CDK1*	NM_001786.4	CTG GCC ACA CTT CAT TAT TGG	GCA CCA TAT TTG CTG AAC TAG C
*CXCL6*	NM_002993.3	TAG TGG TCA AGA GAG GGT TCG	GAG GGA TGA ATG CAG ATA AAG G
*CX3CL1*	NM_002996.3	CGT CAA AGG GAA CCT CTA ACC	CTT GAC CAT TCT CCA CCT TCC
*E2F1*	NM_005225.2	CGT TGG TGA TGT CAT AGA TGC	CAC TGA ATC TGA CCA CCA AGC
*FANCI*	NM_001113378.1	TTT AAC AAG GTG TCC ACA CAG C	CTC TTC TCA GGC AAC CCT ACC
*HIST1H1D*	NM_005320.2	CTC CTT AGA AGC TGC CAC TGC	TCC ACT TGC TCC TAC CAT TCC
*HIST1H1E*	NM_005321.2	GCC TTC TTG TTG AGT TTG AAG G	GAC GTG GAG AAG AAC AAC AGC
*HIST1H2AH*	NM_080596.2	CTG GAT ATT GGG CAA GAC ACC	ACA AGA AGA CCC GTA TCA TCC
*HIST1H2BO*	NM_003527.4	GAG CTG GTG TAC TTG GTG ACG	CTG GCG CAT TAC AAC AAG C
*HIST1H2I*	NM_003525.1	ACT TGG AGC TGG TGT ACT TGG	ACT TCC AGG GAG ATC CAA AGC
*KIF11*	NM_004523.3	CTG ATC AAG GAG ATG TTC ACG	TGG AAC AGG ATC TGA AAC TGG
*KIF15*	NM_020242.1	TGA AGA AAG CTC CTT GTC AGC	GAC CAA ACA GCA GGA AGA GC
*MYBL2*	NM_002466.3	GAG GCT GGA AGA GTT TGA AGG	CTC TGG CTC TTG ACA TTG TGG
*NOS3*	NM_000603.4	GTC CAG GAA GAA GGT GAG AGC	CAG TAG AGC AGC TGG AGA AGG
*NUF21*	NM_145697.2	CAC TTC CAA CTG ACA TGA AGG	TGA AAG ATA CGG TCC AGA AGC
*SELE*	NM_000450.2	GTC TTG GTC TCT TCA CCT TTG C	AAA GAC TCA GTG TTC CCT TTC C
*VCAM1*	NM_001078.3	ACT TTG ACT TCT TGC TCA CAG C	CTG TGC AAA TCC TTG ATA CTG C

### Rodent IA models, histological analysis, and immunohistochemistry of induced IAs

All of the following experiments complied with the National Institutes of Health Guide for the Care and Use of Laboratory Animals and were approved by the Institutional Animal Care and Use Committee of the Kyoto University Graduate School of Medicine.

Sprague-Dawley rats were purchased from Japan SLC (Shizuoka, Japan). To induce IA, male 7-week-old rats were subjected to ligation of the left carotid artery and ligation of the left renal artery under general anesthesia by intraperitoneal injection of pentobarbital sodium (50 mg/kg). Animals were fed a special chow containing 8 % sodium chloride and, in some experiments, 0.12 % 3-aminopropionitrile (Tokyo Chemical Industry, Tokyo, Japan), an inhibitor of lysyl oxidase that catalyzes cross-linking of collagen and elastin. The procedure to induce IAs (defined as “aneurysm induction”) was designed to increase the local hemodynamic stress on the right bifurcation site of the anterior cerebral artery and olfactory artery a contralateral side of carotid ligation [4, 25, 26]. For histological analyses, animals were deeply anesthetized by intraperitoneal injection of a lethal dose of pentobarbital sodium and transcardinally perfused with a fixative, 4 % paraformaldehyde. Then, the circle of Willis including IA lesion was dissected out and serial frozen sections of 5-μm thickness were prepared. For immunohistochemistry, after blocking with 3 % donkey serum (Jackson ImmunoResearch, Baltimore, MD), the sections were incubated with primary antibodies followed by incubation with secondary antibodies conjugated with fluorescent dye (Jackson ImmunoResearch). Finally, immunofluorescence images were acquired with a confocal fluorescence microscope system (Lsm710; Carl Zeiss Microscopy GmBH, Gottingen, Germany). The following primary antibodies were used: mouse monoclonal anti-smooth muscle alpha actin antibody (#MS113; Thermo Scientific, Waltham, MA), goat polyclonal anti-MCP-1 antibody (#sc-1785; Santa Cruz Biotechnology, Dallas, TX), goat polyclonal anti-CX3CL1 antibody (#AF537; R&D Systems, Minneapolis, MN).

### Detection of proliferative cells in rat intracranial arteries

To detect proliferative cells *in vivo*, rats were given an intraperitoneal injection of 5-ethynyl-2′-deoxyuridine (EdU, 80 mg/kg) and, after 24 h, a specimen of intracranial arteries was prepared as described above. EdU intercalated in genome was detected and visualized by click reaction using Alexa488-conjugated azide according to the manufacturer’s instruction (Click-iT EdU Imaging Kits, Life Technologies).

### Statistics

Data are shown in mean ± SEM. Statistical comparisons between more than two groups were conducted using the Kruskal-Wallis test followed by post-hoc Dunn’s test. *P* less than 0.05 was considered statistically significant.

## Results

### Calculation of wall shear stress

**Fig. 1:**
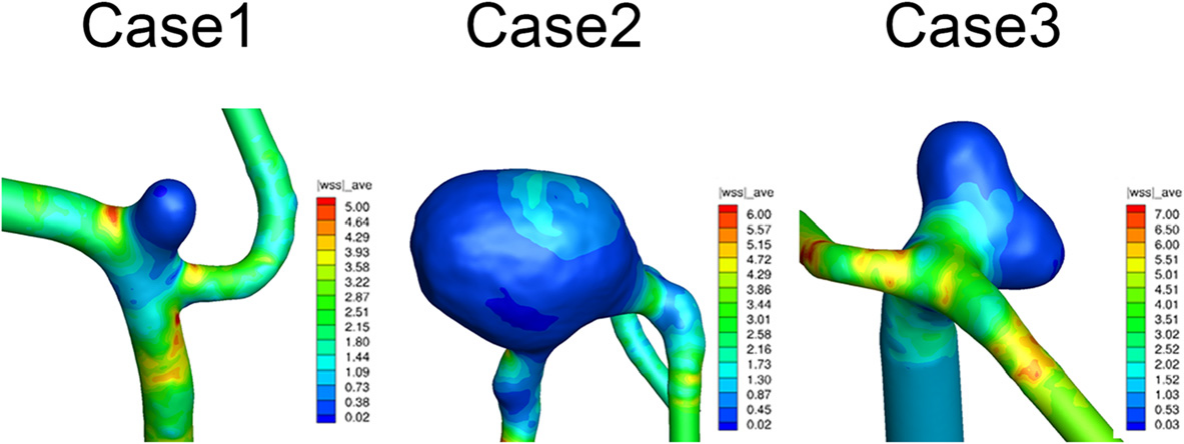
CFD analysis of intracranial aneurysm from three human patients. The value of wall shear stress (WSS) is indicated in the color phase

Three patients with unruptured IAs were enrolled in the present study. WSS loaded on the dome of each IA and the corresponding parent artery was calculated by CFD analysis (Fig. 1). Mean WSS calculated from the three cases were 0.017 Pa (±0.0027, *n* = 3) at the minimum value in the dome of IAs or 6.50 Pa (±0.66, *n* = 3) in the neck portion of IAs, respectively. Notably, WSS in the dome of IAs were strikingly lower compared with those in the parent artery (3.0 Pa), which is consistent with previous reports [12, 13].

### Change of gene expression profile

To verify the effect of each WSS on gene expression, a primary culture of ECs from human carotid artery was cultured and shear stress corresponding to the value (3.0 Pa, 0.05 Pa) obtained in CFD was loaded on these cells for 24 h. In addition, because some studies have demonstrated the presence of turbulent flow in addition to low WSS at the rupture point of IAs in human cases [14, 15], cultured ECs were loaded with the turbulent flow in combination with low WSS. Then, after the quality of purified RNA from the stimulated cells was confirmed by RNA analyzer (RIN > 9.50), the gene expression profile was obtained by RNA sequence–based CAGE analyses. The read count, which was successfully mapped to the reference genome using the MOIRAI workflow system [27], was around 10,000,000, enough for further analyses (Table 2). Then, profiling differentially expressing genes (peaks in RNA sequence mapped to the reference genome) between experimental groups was obtained by a pipeline, RECLU [28]. Here, the RECLU pipeline extracted two types of clusters, top peaks and bottom ones [28]. Briefly, top peaks or bottom ones represent the narrowest or broadest reproducible peaks in an RNA sequence [28]. Upregulated or downregulated peaks in a 0.05 Pa–loaded group compared with those in a 3.0 Pa–loaded group were identified; the total number was around 300 (Table 3). In another comparison, differentially expressed genes were also identified (Table 3). For the GO analyses, GO terms related with cell division/proliferation, such as nucleosome, mitotic cell cycle, and DNA replication, were highly overrepresented in the 0.05 Pa + turbulent flow–loaded group compared with the 3.0 Pa-loaded group (Table 4). Indeed, overexpressed genes in the 0.05 Pa + turbulent flow–loaded group included those related with cell division and proliferation (Table 5), which is consistent with a previous study that turbulent flow enhances proliferation of ECs [29]. Underrepresented terms in this comparison included IL-33 receptor activity and IL-1 receptor activity, but the *p* value was much less compared with that of the overrepresented terms (Table 4). In comparisons between the 0.05 Pa-loaded group and the 3.0 Pa-loaded group or 0.05 Pa + turbulent flow–loaded group and 0.05 Pa-loaded group, GO terms related with cell division/proliferation, such as mitosis, were flagged as overrepresented in the 0.05 Pa-loaded (Table 6) or 0.05 Pa + turbulent flow–loaded group (Table 7), respectively, suggesting that cell division/proliferation was enhanced in a low WSS condition and further amplified by turbulent flow. In pathway analyses, biological processes consistently related with cell division/proliferation, such as telomere maintenance, mitotic cell cycle, and DNA replication, were overrepresented in a set of genes from the low WSS-loaded group compared with that of the 3.0 Pa-loaded group (Table 8).

**Table 2:** Read count obtained in RNA-sequence-based CAGE analysis

	sample No.	Mapped Read Count	rRNA read count
0.05Pa	1	11,216,634	2,265,251
	2	8,936,578	2,108,157
3.0Pa	1	11,293,958	1,776,765
	2	9,160,492	2,264,215
0.05Pa + TF	1	6,765,681	1,171,547
	2	9,547,702	1,795,395

*TF* turbulent flow

**Table 3:** Number of differentially expressed peaks between each comparison

	up-regulated peaks		down-regulated peaks	
	Top peaks	Bottom peaks	Top peaks	Bottom peaks
3.0Pa vs. 0.05Pa	314	331	261	385
3.0Pa vs. 0.05Pa + TF	604	562	496	395
0.05Pa vs. 0.05Pa + TF	78	214	74	148

*TF* turbulent flow

**Table 4:** GO term analysis of differentially expressing genes between 3.0Pa-loaded and 0.05Pa + turbulent flow-loaded group

Over-presented term in 0.05Pa + turbulent flow-loaded group			
Accession	Term	*P*-value	FDR
GO:0000786	nucleosome	7.7E-54	3.0E-51
GO:0006334	nucleosome assembly	6.4E-53	1.3E-50
GO:0000278	mitotic cell cycle	3.8E-26	5.1E-24
GO:0007067	mitosis	9.7E-26	9.6E-24
GO:0046982	protein heterodimerization activity	9.7E-24	7.7E-22
GO:0005654	nucleoplasm	5.6E-19	3.7E-17
GO:0060968	regulation of gene silencing	8.7E-15	4.7E-13
GO:0003677	DNA binding	9.5E-15	4.7E-13
GO:0005819	spindle	8.5E-14	3.7E-12
GO:0000775	chromosome, centromeric region	3.5E-13	1.4E-11
GO:0005634	nucleus	4.8E-13	1.7E-11
GO:0000777	condensed chromosome kinetochore	9.2E-13	3.1E-11
GO:0034080	CENP-A containing nucleosome assembly at centromere	5.9E-12	1.8E-10
GO:0007059	chromosome segregation	1.6E-11	4.5E-10
GO:0006260	DNA replication	1.1E-10	2.9E-09
GO:0030496	midbody	1.6E-10	3.9E-09
GO:0031536	positive regulation of exit from mitosis	2.1E-10	4.8E-09
GO:0000922	spindle pole	3.6E-10	7.8E-09
GO:0005876	spindle microtubule	1.9E-09	4.0E-08
GO:0006281	DNA repair	2.5E-09	5.0E-08
Under-presented term in 0.05Pa + turbulent flow-loaded group			
Accession	Term	*P*-value	FDR
GO:0002113	interleukin-33 binding	2.9E-07	5.9E-05
GO:0002114	interleukin-33 receptor activity	2.9E-07	5.9E-05
GO:0043032	positive regulation of macrophage activation	7.9E-07	1.1E-04
GO:0090197	positive regulation of chemokine secretion	1.0E-06	1.1E-04
GO:0004908	interleukin-1 receptor activity	1.5E-06	1.2E-04
GO:0002826	negative regulation of T-helper 1 type immune response	1.7E-06	1.2E-04
GO:0032754	positive regulation of interleukin-5 production	3.6E-05	2.1E-03
GO:0070698	type I activin receptor binding	5.9E-05	3.0E-03
GO:0071310	cellular response to organic substance	1.1E-04	4.9E-03
GO:0030617	inhibitory cytoplasmic mediator activity	1.2E-04	4.9E-03

*FDR* false discovery rate

**Table 5:** List of over-expressed genes in 0.05Pa + turbulent flow-loaded group compared with 3.0Pa-loaded group

Gene Symbol	Gene Accession	Log Fold Change	*P*-value	Peaks
PRND	ENST00000305817	5.780	6.90E-14	BOTTOM
LTB	ENST00000429299	6.170	3.80E-10	TOP
KIF15	ENST00000438321	6.110	8.80E-10	TOP
KIF20A	ENST00000508792	8.290	1.40E-09	TOP
CENPA	ENST00000233505	6.020	1.60E-09	TOP
NCAPH	ENST00000240423	7.770	1.80E-07	TOP
KCNAB1	ENST00000389634	7.430	4.60E-06	TOP
LRRC17	ENST00000498487	7.270	6.00E-06	TOP
ASPM	ENST00000294732	7.040	4.00E-05	TOP
CDC45	ENST00000404724	6.890	0.00011	TOP
RP11-322E11.5	ENST00000593122	6.770	0.00021	TOP
HIST1H2AH	ENST00000377459	6.710	0.00026	TOP
SPC24	ENST00000423327	6.830	0.0004	TOP
FAM64A	ENST00000572447	6.510	0.0018	TOP
MCM7	ENST00000489841	6.360	0.0029	TOP
HIST1H1B	ENST00000331442	6.100	0.0047	BOTTOM
DAB2IP	ENST00000436835	6.100	0.0047	BOTTOM
RRM2	ENST00000459969	6.190	0.0052	TOP
DEPDC1B	ENST00000453022	6.100	0.0082	TOP
EXO1	ENST00000423131	6.100	0.009	TOP
PDLIM3	ENST00000284770	6.000	0.01	TOP
CENPM	ENST00000402420	6.000	0.015	TOP
PAK1	ENST00000528592	6.000	0.015	TOP
ME3	ENST00000526834	5.900	0.015	TOP
HIST1H2BI	ENST00000377733	5.780	0.017	BOTTOM
TTK	ENST00000509313	5.900	0.019	TOP
ITM2B	ENST00000463839	6.100	0.02	TOP
ERCC6L	ENST00000373657	5.790	0.024	TOP
KIT	ENST00000514582	5.790	0.024	TOP
CENPA	ENST00000419525	5.900	0.025	TOP

**Table 6:** Over-presented term in 0.05Pa -loaded group compared with 3.0Pa-loaded group in GO term analysis

Accession	Term	*P*-value	FDR
GO:0000786	nucleosome	3.5E-27	4.9E-25
GO:0006334	nucleosome assembly	1.5E-24	1.1E-22
GO:0046982	protein heterodimerization activity	1.3E-14	6.0E-13
GO:0060968	regulation of gene silencing	4.9E-10	1.7E-08
GO:0000278	mitotic cell cycle	3.4E-09	9.6E-08
GO:0003677	DNA binding	4.7E-09	1.1E-07
GO:0005654	nucleoplasm	7.3E-09	1.5E-07
GO:0031145	anaphase-promoting complex-dependent proteasomal ubiquitin-dependent protein catabolic process	1.9E-06	3.3E-05
GO:0006260	DNA replication	1.3E-05	1.7E-04
GO:0010994	free ubiquitin chain polymerization	1.3E-05	1.7E-04
GO:0000083	regulation of transcription involved in G1/S transition of mitotic cell cycle	1.4E-05	1.7E-04
GO:0051437	positive regulation of ubiquitin-protein ligase activity involved in mitotic cell cycle	2.8E-05	3.3E-04
GO:0051439	regulation of ubiquitin-protein ligase activity involved in mitotic cell cycle	3.3E-05	3.4E-04
GO:0043565	sequence-specific DNA binding	3.4E-05	3.4E-04
GO:0031536	positive regulation of exit from mitosis	4.6E-05	4.3E-04
GO:0007067	mitosis	5.4E-05	4.7E-04
GO:0008054	cyclin catabolic process	6.6E-05	5.4E-04
GO:0000281	mitotic cytokinesis	7.2E-05	5.6E-04
GO:0005634	nucleus	8.0E-05	5.9E-04
GO:0034501	protein localization to kinetochore	1.0E-04	7.1E-04

*FDR* false discovery rate

**Table 7:** Over-presented term in 0.05Pa + turbulent flow-loaded group compared with 0.05Pa-loaded group in GO term analysis

Accession	Term	*P*-value	FDR
GO:0000775	chromosome, centromeric region	9.3E-07	3.6E-05
GO:0007067	mitosis	9.9E-07	3.6E-05
GO:0000786	nucleosome	1.9E-06	4.7E-05
GO:0000278	mitotic cell cycle	8.4E-06	1.5E-04
GO:0031536	positive regulation of exit from mitosis	1.2E-05	1.7E-04
GO:0030496	midbody	1.8E-05	2.1E-04
GO:0006334	nucleosome assembly	2.3E-05	2.4E-04
GO:0000079	regulation of cyclin-dependent protein kinase activity	6.5E-05	5.9E-04
GO:0007059	chromosome segregation	1.4E-04	1.2E-03
GO:0051382	kinetochore assembly	2.0E-04	1.5E-03
GO:0000070	mitotic sister chromatid segregation	2.8E-04	1.9E-03
GO:0000922	spindle pole	3.2E-04	1.9E-03
GO:0051301	cell division	3.7E-04	2.1E-03
GO:0000086	G2/M transition of mitotic cell cycle	8.6E-04	4.5E-03
GO:0007094	mitotic spindle assembly checkpoint	9.3E-04	4.5E-03
GO:0005164	tumor necrosis factor receptor binding	1.3E-03	6.0E-03
GO:0000777	condensed chromosome kinetochore	2.9E-03	1.2E-02
GO:0007165	signal transduction	3.8E-03	1.5E-02
GO:0046982	protein heterodimerization activity	3.9E-03	1.5E-02
GO:0000910	cytokinesis	4.1E-03	1.5E-02

*FDR* false discovery rate

**Table 8:** Over-represented pathways in 0.05Pa-loaded or 0.05Pa + turbulent flow-loaded group compared with 3.0Pa-loaded group

Comparison	Peaks	REACTOME ID	Pathway	*P*-value	FDR
3.0Pa < 0.05Pa + TF	Top peaks	REACT_7970	Telomere Maintenance	7.53E-12	1.36E-10
		REACT_8017	APC-Cdc20 mediated degradation of Nek2A	3.74E-03	2.32E-02
		REACT_152	Cell Cycle, Mitotic	3.86E-03	2.32E-02
	Bottom peaks	REACT_7970	Telomere Maintenance	2.67E-14	4.01E-13
		REACT_152	Cell Cycle, Mitotic	8.97E-03	6.73E-02
3.0Pa < 0.05Pa	Top peaks	REACT_7970	Telomere Maintenance	5.21E-08	5.21E-07
		REACT_383	DNA Replication	2.24E-02	1.12E-01
		REACT_1538	Cell Cycle Checkpoints	8.86E-02	2.48E-01
		REACT_6850	Cdc20:Phospho-APC/C mediated degradation of Cyclin A	1.20E-01	2.48E-01
		REACT_152	Cell Cycle, Mitotic	1.35E-01	2.48E-01
		REACT_8017	APC-Cdc20 mediated degradation of Nek2A	1.49E-01	2.48E-01
	Bottom peaks	REACT_7970	Telomere Maintenance	1.16E-06	6.96E-06
		REACT_152	Cell Cycle, Mitotic	1.03E-02	3.09E-02
		REACT_383	DNA Replication	3.15E-02	6.30E-02

*FDR* false discovery rate

In addition to genes located at the head of the list, which included many genes related with cell division or proliferation (Table 5), upregulated genes in the 0.05 Pa or 0.05 Pa + turbulent flow–loaded group included chemoattractants or adhesion molecules for inflammatory cells. Next, we focused on these chemoattractants and adhesion molecules because inflammatory cells, especially macrophages, play a crucial role in IA formation/progression [10, 11, 30]. In the low WSS condition (0.05 Pa), expression of *CX3CL1*, a chemoattractant for monocytes, and *VCAM-1* were induced compared with the 3.0 Pa-loaded group (Table 9). Presence of turbulent flow concomitant with low WSS further augmented the expression of chemoattractants and adhesion molecules such as *CCL2* (gene for MCP-1), a chemoattractant for macrophages, *CXCL6*, a chemoattractant for granulocytes, and *SELE* (gene for E-selectin) (Table 9). These results suggest that turbulent flow with concomitant low WSS induces chemoattractants and adhesion molecules for inflammatory cells in ECs that in turn recruit cells in lesions to evoke inflammation.

**Table 9:** Up-regulation of chemoattractants and adhesion molecules for inflammatory cells in 0.05Pa or 0.05Pa + turbulent flow-loaded group

3.0Pa < 0.05Pa						
Symbol	Gene Accession	Start	End	Log Fold Change	*P*-value	Peaks
CX3CL1	ENST00000006053	57406348	57406458	3.20	2.9E-03	BOTTOM
CX3CL1	ENST00000563383	57406400	57406402	3.69	2.1E-02	TOP
VCAM1	ENST00000370119	101185296	101185298	4.89	1.7E-03	TOP
VCAM1	ENST00000370119	101185284	101185300	4.37	6.9E-06	BOTTOM
0.05Pa < 0.05Pa + turbulent flow						
Symbol	Gene Accession	Start	End	Log Fold Change	*P*-value	Peaks
CCL2	ENST00000225831	32582302	32582304	2.27	2.4E-04	TOP
CCL2	ENST00000225831	32582222	32582386	2.06	1.2E-06	BOTTOM
CXCL1	ENST00000509101	74735046	74735111	1.45	1.2E-02	BOTTOM
CXCL6	ENST00000515050	74702302	74702396	1.87	2.6E-02	BOTTOM
ICAM1	ENST00000423829	10381756	10381818	1.39	7.5E-03	BOTTOM
ICAM2	ENST00000581417	62084077	62084249	1.38	5.5E-03	BOTTOM
SELE	ENST00000367779	169703219	169703222	2.95	7.0E-03	BOTTOM
VCAM1	ENST00000370119	101185275	101185302	1.33	2.6E-02	BOTTOM
3.0Pa < 0.05Pa + turbulent flow						
Symbol	Gene Accession	Start	End	Log Fold Change	*P*-value	Peaks
CCL2	ENST00000225831	32582302	32582304	2.08	2.6E-03	TOP
CCL2	ENST00000225831	32582222	32582386	1.94	4.4E-04	BOTTOM
CCL2	ENST00000225831	32582222	32582224	2.15	4.5E-02	TOP
CX3CL1	ENST00000563383	57406400	57406402	2.19	4.7E-04	TOP
CX3CL1	ENST00000006053	57406348	57406402	2.03	1.7E-04	BOTTOM
CXCL11	ENST00000306621	76957183	76957237	2.89	1.4E-02	TOP
CXCL6	ENST00000515050	74702302	74702396	2.11	1.6E-02	BOTTOM
SELE	ENST00000367779	169703220	169703222	3.69	1.5E-02	TOP
SELE	ENST00000367779	169703219	169703222	2.94	2.8E-03	BOTTOM
VCAM1	ENST00000370119	101185275	101185302	1.33	2.6E-02	BOTTOM

### RT-PCR analyses of genes differentially expressed under each WSS condition

**Fig. 2:**
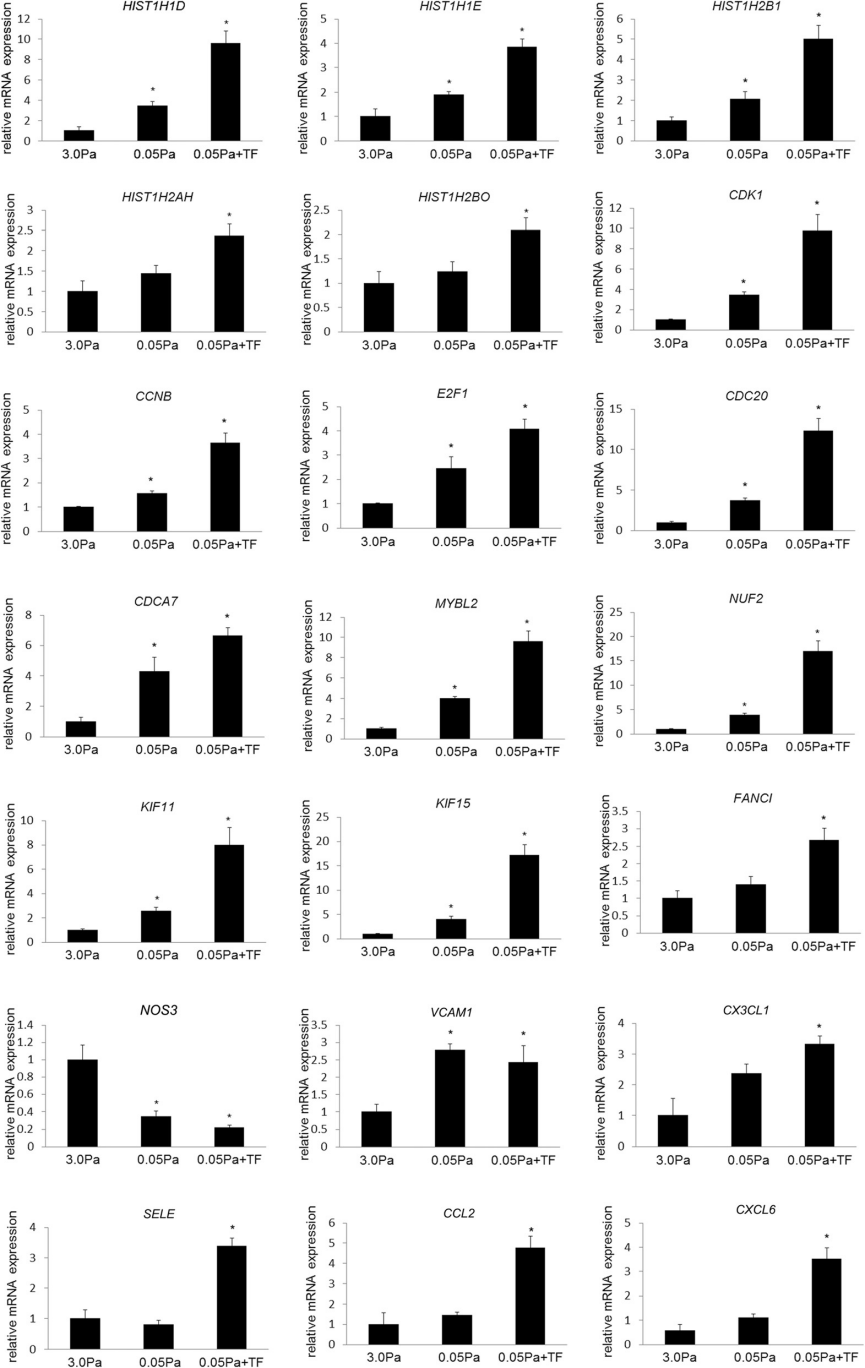
Induction of genes in cultured endothelial cells (ECs) loaded on low WSS and turbulent flow. RNA was purified from cultured ECs loaded on 3.0 Pa, 0.05 Pa, or 0.05 Pa with turbulent flow (TF), and gene expressions were analyzed by RT-PCR analysis. All *bars* indicate mean ± SEM (*n* = 3). ^*^ *p* < 0.05 compared with 3.0 Pa-loaded group

To confirm results of the gene expression profile obtained by the RNA sequence–based CAGE analysis, we examined the expression of selected genes by RT-PCR and found that the results could be reproduced (Fig. 2). For example, genes related to cell proliferation/division, such as histone subunit genes (*HIST1HID, etc.*), cyclin or cyclin-dependent kinase (*CDK1, CCNB*), transcription factor regulating cell cycle (*E2F1*), genes regulating cell division (*CDC20, CDCA7*), the nuclear protein promoting cell cycle (*MYBL2*), gene regulating chromatin segregation (*NUF2*), kinesins that transport materials in cytoplasm (*KIF11, KIF15*), or gene encoding protein stabilizing DNA (*FANCI*) were induced in ECs loaded on low WSS (Fig. 2). Interestingly, *NOS3* (eNOS) expression was downregulated under low WSS conditions in RT-PCR (Fig. 2), suggesting that there is a dysfunction of ECs in this WSS condition, as demonstrated by previous studies in which there was a loss of gap junction during IA formation [31] or a decrease of eNOS staining in the dome of IAs [32]. We also confirmed that the genes for chemoattractants and adhesion molecules, *VCAM1, CX3CL1, SELE, CCL2, CXCL6,* were induced under low WSS or low WSS with concomitant turbulent flow condition (Fig. 2) during the RNA sequence analysis (Table 9). Again, we noted the remarkable contribution of turbulent flow in addition to concomitant low WSS to induction of these genes.

### Proliferative status of intracranial arterial walls during IA formation

**Fig. 3:**
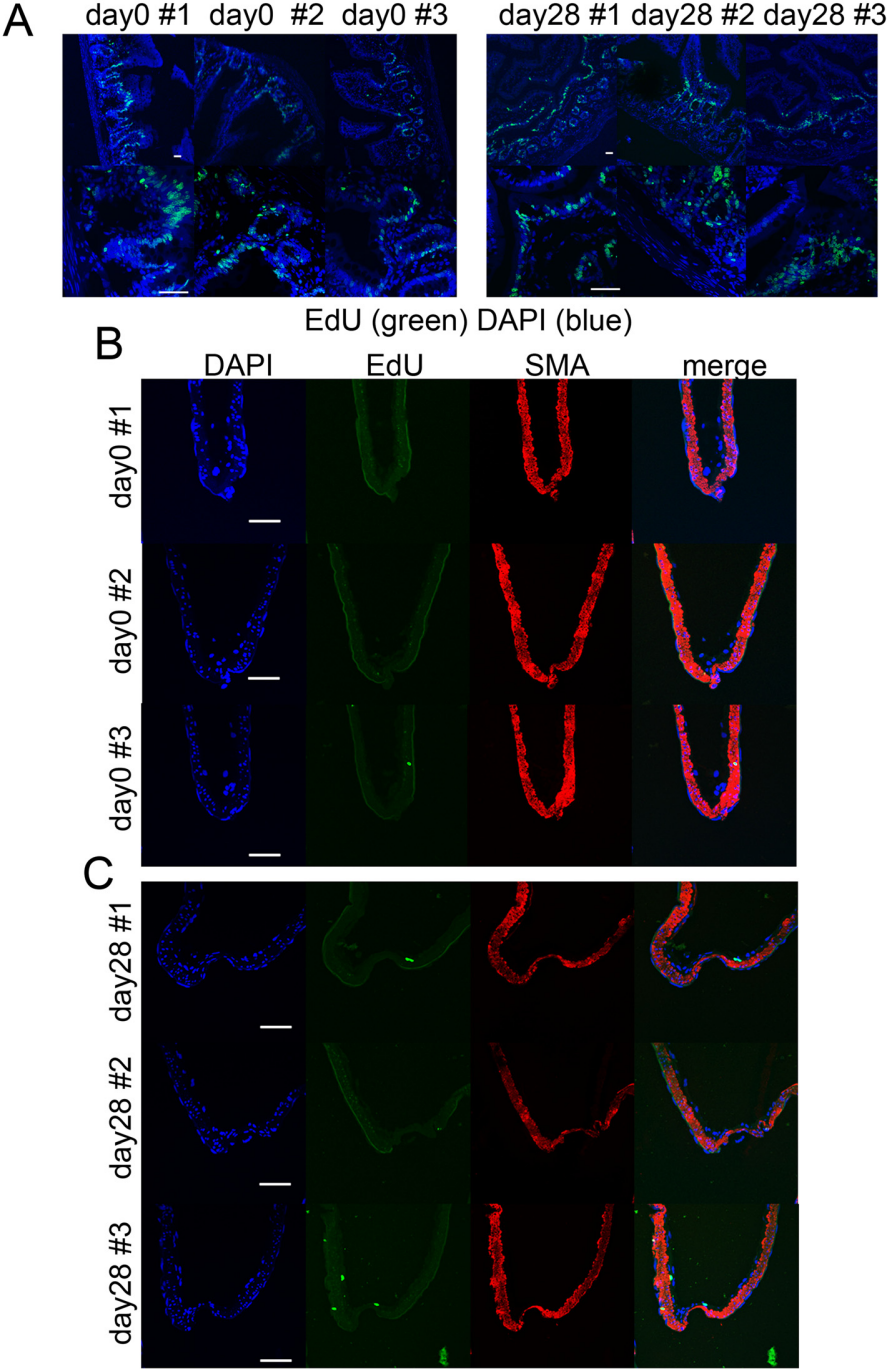
Detection of proliferative cells in intracranial aneurysm lesions of rat. **a**, Proliferative cells in the small intestine of rats after intraperitoneal injection of EdU (80mg/kg) were detected by click reaction (green color). Merged images with nuclear staining by DAPI are shown. Magnified images are shown in the lower panels. Scale bar = 50μm. **b** and **c**, Proliferative cells in intracranial arterial walls of rats before (day 0, **b**) or after (day 28, **c**) aneurysm induction were labeled after intraperitoneal injection of EdU. Immunostaining for α-smooth muscle actin (SMA) is shown to visualize the arterial walls. Scale bar = 50μm

In response to the results of an *in vitro* study that showed pathways related with proliferation of ECs are overrepresented under low WSS and concomitant turbulent flow, we examined the proliferation of cells in intracranial arterial walls *in vivo*. In this experiment, we used Edu, which was intercalated in the genome during replication of proliferative cells as an alternative of thymidine, to detect proliferative cells in rat tissue. In the small intestine, which was used as a positive control, proliferative cells incorporated with EdU were detected as Alexa488-positive cells in all rats that were examined (Fig. 3a), confirming the proper procedure to detect proliferative cells *in vivo*. In intracranial arterial walls before and after IA induction, a small number of Alexa488-positive proliferative cells were identified, but no signaling was detectable in the endothelial cell layer (Fig. 3b and c), suggesting that ECs in intracranial arteries do not actively proliferate.

### Temporal sequence of MCP-1 and CXCL3 expression in IA lesions *in vivo*

To verify the *in vivo* relevance of our *in vitro* study, we examined the temporal sequence of MCP-1 and CX3CL1 expression in the rat IA model. We selected these two molecules because they could recruit macrophages, which serve as major inflammatory cells in lesions both in human and animal models [33, 34]. In addition, MCP-1–mediated recruitment of macrophages plays a crucial role in IA formation/progression via the secretion of a variety of pro-inflammatory factors because genetic deletion or inhibition of MCP-1 has been shown to almost completely suppress IA formation in animal models [11, 30].

**Fig. 4:**
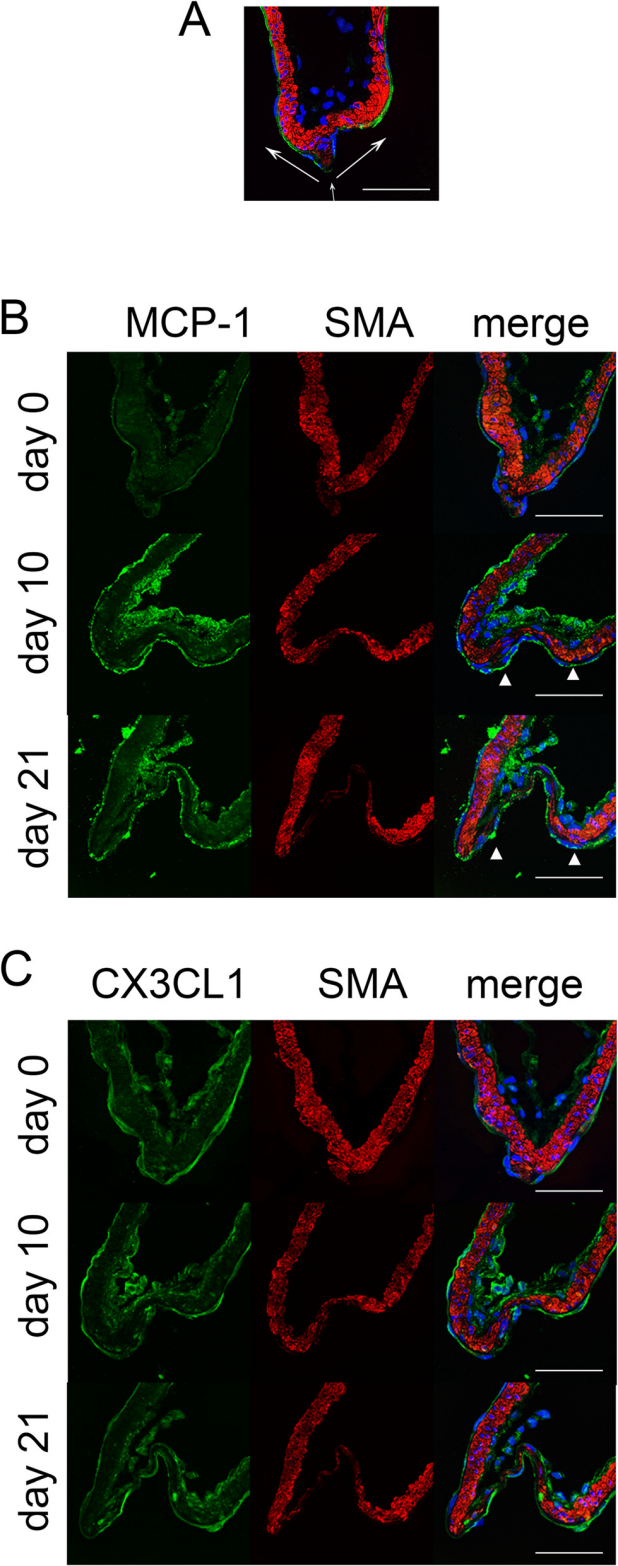
Induction of MCP-1 in the endothelial cell layer during intracranial aneurysm formation in rats. **a**, Representative image to show the anatomical structure of the bifurcation site of the intracranial artery. A merged image of immunohistochemistry for smooth muscle marker, α-smooth muscle actin (SMA, red), an endothelial cell marker, CD31 (green), and nuclear staining by DAPI (blue) is shown. *White arrows* indicate the direction of blood flow. Scale bar = 50μm. **b** and **c**, Expression of MCP-1 (**b**) and CX3CL1 (**c**) in the endothelial cell layer of arterial walls during IA formation. Immunohistochemistry for MCP-1 (green in **b**), CX3CL1 (green in **c**), SMA (red), and for the merged images with DAPI (blue) are shown. Scale bar = 50μm

MCP-1 expression was only weakly detected in ECs of intracranial arteries before induction (Fig. 4a and b). Its intensity in immunostaining increased and spread to adventitia of arterial walls after IA induction, which was consistent with our previous study [30]. Importantly, expression of MCP-1 in ECs of IA walls was sustained, not decreased, during IA formation (Fig. 4a and b), suggesting the *in vivo* relevance of *in vitro* study.

In the case of CX3CL1, expression was persistently detectable in intracranial arteries, including endothelial cell layer, from day 0 to day 21 of IA induction (Fig. 4c). However, the signal intensity did not change during IA formation (Fig. 4c), which was consistent with our previous study of the gene expression profile of ECs from IA lesions [35], indicating the independence of CX3CL1 in IA formation.

## Discussion

Recent studies have provided experimental evidence that long-lasting inflammatory responses in intracranial arteries play a crucial role in IA formation [10]. During this process, MCP-1-mediated macrophage recruitment and macrophage-evoked inflammation are critical for IA formation because of the genetic deletion of MCP-1, inhibition of MCP-1 by a dominant negative form (7-ND), or pharmacological depletion of macrophages that remarkably suppress IA formation [11, 30]. MCP-1 is induced in ECs in intracranial arteries under a high WSS condition loaded at an early stage of IA formation [30]. Although shear stress dramatically changes during IA progression, from high WSS at an early stage [7, 8] to low WSS, sometimes with turbulent flow, at a later stage [12–14], MCP-1 expression is sustained in ECs of IA lesions induced in a rat model both by immunostaining [30] and RT-PCR analysis [35]. Consistently, MCP-1 expression is also detected in ECs of human IA walls [30] in which low WSS is present. The current experiment addressed this issue and provided experimental evidence linking low WSS with concomitant turbulent flow and MCP-1 expression in ECs of IA walls.

MCP-1 expression under a low WSS stress condition is presumably critical for macrophage infiltration in pathological conditions, as observed in IA walls, to exacerbate inflammatory responses leading to progression of IAs. This is due to the fact that a low WSS condition facilitates adhesion of macrophages to ECs expressing MCP-1, but a high WSS condition interferes with adhesion. Furthermore, the present study again highlights the importance of MCP-1 over the entire period of IA formation/progression because induction of another chemoattractant for macrophages, CX3CL1, is not obvious during IA formation. Among genes induced under a low WSS condition in ECs, VCAM-1 [5] and E-selectin [36] induction in IA lesions, including ECs in the dome of a rat model, have been reported, suggesting the *in vivo* relevance of the present study. Consistent with our RNA-sequence and RT-PCR analyses that VCAM-1 can be induced under a low WSS condition, VCAM-1 expression in ECs in the dome of IAs induced in a rat model has been demonstrated [5]. However, the contribution of this molecule to IA formation and progression is not clear. E-selectin can also be induced in ECs in the dome of IAs during IA formation [36]. However, because inhibition of E-selectin expression by cimetidine failed to suppress infiltration of macrophages in lesion and IA formation [36], the contribution of E-selectin to IA formation could be interpreted as negative. Intriguingly, the present study clearly demonstrated the importance of concomitant turbulent flow with low WSS in the exacerbation of macrophage infiltration via MCP-1 induction. Since the media of IA walls becomes gradually thinner and smooth muscle cells in media are shed during IA progression, these histopathological changes of IA walls and MCP-1 induction under a low WSS condition remarkably facilitates macrophage infiltration and the resultant exacerbation of inflammation and tissue destruction that presumably leads to rupture of IAs. Indeed, massive macrophage infiltration of lesions has been reported in ruptured human IAs, but not in unruptured ones, suggesting the role of macrophages in the rupture of IAs. However, the rupture of IAs and resultant inflammatory response itself may greatly increase macrophage infiltration [37–39]. This hypothesis goes hand-in-hand with the observation that turbulent flow with low WSS can be detected at the rupture point of IAs [14, 15], although contribution of abnormally high WSS to the rupture of IAs is also presumed [16].

The present study includes several limitations. First, there are potential limitations related to CFD. Specifically, blood was treated as a Newtonian fluid and the wall was assumed to be rigid in our CFD simulations. These simplified properties might lead to a different state compared with the *in vivo* state. However, the effect of a non-Newtonian property on large artery hemodynamics is believed to be small [40, 41]. In addition, it has been reported that the overall feature of WSS distribution does not considerably change when incorporating wall deformation [42]. Thus, our CFD simulations provide a reasonable WSS approximation of the *in vivo* state. Another limitation is that our sample size was small (3 cases of IA). We infer that the present low-WSS characteristics in the dome are consistent among clinical cases, as demonstrated by previous studies [12, 13, 43]. However, future studies will have to allow for more realistic estimation of WSS, considering the wide range of geometries that intracranial aneurysms can have. The IA rat model used in the present study also has some intrinsic limitations. IAs induced in a rat model are not saccular with a narrow neck, but rather have a mountain-like shape with a wide neck. Further, this model does not allow for assessment of enlargement in the same animal due to the small size of the lesions nor rupture because of low incidence of spontaneous rupture [25]. Therefore, we exclusively analyzed the enlargement of IAs in animal models, and from this reference point we have provided evidence geared toward developing therapeutic drugs to prevent the enlargement of IAs. Here, because statins suppressed enlargement of IAs in a rat model [44–46] and usage of this class of drugs reduced the relative risk of subarachnoid hemorrhage in a Japanese case-control study [47], some drugs preventing progression of IAs in animal models should be considered for human IAs to prevent rupture. However, because approximately one third of human IAs rupture but the remaining never rupture [48], there may be different pathological processes at work. In addition, we used primary culture of ECs from the carotid artery, which is an extra-cranial artery, because we could not obtain ECs from human intracranial artery. Differences in the origins of ECs may influence results and, therefore, we need to pay careful attention when analyzing data. One final major limitation of the present study is that we could not reconstitute whole arterial walls, especially the physical or chemical interaction with adjacent cells or structures such as medial smooth muscle cells or internal elastic lamina. Indeed, turbulent flow concomitant with low WSS greatly activates proliferation of ECs *in vitro*, but not in IA walls *in vivo*. To examine the detailed interaction of hemodynamic forces with arterial walls, including ECs leading to pathological situations, organ culture using whole arterial walls may be necessary.

## Conclusions

In the present study, we used primary culture of ECs loaded on shear stress, RNA sequencing of these cells, bioinformatic analysis of RNA-sequencing data, and verification of results from *in vitro* data using a rat model of IAs. These techniques showed that low wall shear stress with concomitant turbulent flow induced expressions of chemoattractant and adhesion molecules for macrophages such as MCP-1 in endothelial cells, which could be the mechanism behind sustained macrophage infiltration during IA progression and presumably rupture. In other words, we have clarified that MCP-1 expression is sustained during IA formation/progression independent of flow condition.

## Abbreviations

CAGE: cap analysis of gene expression
CFD: computational fluid dynamic
COX: cyclooxygenase
EC: endothelial cell
GO: gene ontology
IA: intracranial aneurysm
WSS: wall shear stress
